# Iron Deficiency in Newly Referred Patients With Chronic Renal Failure

**DOI:** 10.7759/cureus.61076

**Published:** 2024-05-25

**Authors:** Mineaki Kitamura, Hiroshi Yamashita, Ryoma Kuroki, Haruka Fukuda, Atsushi Sawase, Hiroshi Mukae, Tomoya Nishino

**Affiliations:** 1 Nephrology, Nagasaki University Hospital, Nagasaki, JPN; 2 Nephrology, Nagasaki Habor Medical Center, Nagasaki, JPN; 3 Nephrology, Nagasaki University Graduate School of Biomedical Sciences, Nagasaki, JPN; 4 Respiratory Medicine, Nagasaki University Graduate School of Biomedical Sciences, Nagasaki, JPN

**Keywords:** ischemic heart disease (ihd), erythropoietin, hypoxia-inducible factor prolyl hydroxylase inhibitor, iron deficiency, renal anemia, chronic kidney disease

## Abstract

Addressing iron deficiency is the key to managing anemia in patients with chronic kidney disease (CKD). Erythropoiesis-stimulating agents (ESAs) and hypoxia-inducible factor prolyl-hydroxylase inhibitors (HIF-PHIs) are being prescribed to an increasing number of patients with CKD by primary physicians following the emergence of newer agents for the management of renal anemia. Among the 361 (average age: 76.8±12.1 years; 54.0% males) patients with stages 4 and 5 CKD newly referred to the nephrology department of our hospital between 2018 and 2023 who had evaluable transferrin saturation (TSAT) and ferritin levels, 169 patients (47%) had iron deficiency (ferritin <100 ng/mL or ferritin 100-300 ng/mL with TSAT <20%). The estimated glomerular filtration rate (eGFR), hemoglobin level, TSAT, and median ferritin level were 17.0±7.0 mL/min/1.73 m², 10.8±2.1 g/dL, 27.5±13.1%, and 130 ng/mL, respectively. ESAs, HIF-PHIs, and iron supplements were prescribed to 35 (9.7%), 17 (4.7%), and 35 (9.4%) patients, respectively. No significant differences were observed between the iron indices of the ESA group; however, the serum ferritin levels in the HIF-PHIs group were significantly lower than in those in the no-medication group (P=0.02). Multivariable logistic regression analysis revealed that age, female sex, eGFR, medications for renal anemia, and a history of ischemic heart disease were associated with iron deficiency (P<0.05). Although patients with renal failure tend to exhibit anemia, attention should be paid to iron deficiency anemia in addition to renal anemia, especially in patients with renal failure and a history of ischemic heart disease.

## Introduction

Anemia is frequently observed in patients with chronic kidney disease (CKD), and renal anemia is one of its most noteworthy etiologies. The introduction of new therapeutic agents in recent years has increased interest in the treatment of renal anemia [1]. For instance, in addition to erythropoiesis-stimulating agents (ESAs), a new class of drugs, hypoxia-inducible factor prolyl-hydroxylase inhibitors (HIF-PHIs), is being used for the treatment of renal anemia in patients with CKD, including those who are not on dialysis [2]. The administration of renal anemia agents has increased with the increase in awareness of CKD [3]. However, unlike ESAs, HIF-PHIs are administered orally, which is more convenient and spares the pain associated with ESA injections [3].

Anemia in patients with CKD is multifactorial; thus, the cause of anemia should be elucidated before commencing treatment for renal anemia [1]. Iron deficiency is a major complication in patients with CKD [4]. Several agents are available for the treatment of renal anemia; however, maintaining adequate iron levels is fundamental for ensuring the effectiveness of ESAs and HIF-PHIs [5]. Iron supplementation may be required in some cases to optimize the response to these agents. HIF-PHIs, in contrast to traditional ESAs, act upstream of the erythropoietin production pathway and affect iron metabolism, thereby improving the use of iron for the production of red blood cells [1].

An increasing number of primary physicians are likely to prescribe anti-anemia agents, given the availability of several agents for the treatment of renal anemia and the widespread use of ESAs and HIF-PHIs in primary care facilities in Japan. We hypothesized that some patients with CKD may experience iron deficiency owing to the administration of anti-renal anemia agents, especially HIF-PHIs. Approximately 100 patients with stage 4 or 5 CKD who may have renal anemia are annually referred by primary physicians to our facility, an emergency hospital in Nagasaki City. This study aimed to elucidate the prevalence of iron deficiency among newly referred patients with renal failure at our center and identify the associated factors.

## Materials and methods

Study design

Patients with stage 4 or 5 CKD who were referred to our facility by primary physicians between January 2018 and December 2023 were included in this study. Patients aged <18 years and those whose iron indices (transferrin saturation [TSAT] and serum ferritin levels) were unavailable at the time of the first visit were excluded from the study.

Data collection

Patient characteristics were extracted from the medical records at our hospital. Details regarding the regularly prescribed medications were extracted from the referral letters or medication records. In accordance with the Guidelines for Renal Anemia in Chronic Kidney Disease issued by the Japanese Society for Dialysis Therapy, lower target levels of hemoglobin were defined as 11 g/dL, and iron deficiency was defined as ferritin levels of <100 ng/mL or TSAT of <20% with ferritin levels of 100-300 ng/mL, which is an indication for iron supplementation [6]. A ferritin level of <100 ng/mL with a TSAT of 20% was defined as absolute iron deficiency [7]. The cause of renal failure was determined on the basis of the medical records maintained by the attending physician. “Medications for renal anemia” included ESAs and HIF-PHIs. Patients on oral iron supplementation could include patients with iron deficiency; however, we merely wished to evaluate the iron status at the first visit. Therefore, we differentiated them using serum ferritin levels and TSAT.

Anemia treatment

The prescription data for iron supplementation or medicines for renal anemia at our department were obtained within three months after the first referral.

Statistical analysis

Continuous values are presented as mean ± standard deviations, whereas categorical variables are presented as numbers and percentages. Non-normally distributed data are presented as medians with interquartile ranges (IQR). The chi-square test and Wilcoxon rank-sum test were used to analyze categorical and continuous variables, respectively. The Bonferroni post-hoc correction was used for comparison among the three groups. Multivariable logistic regression analysis for iron deficiency was performed using three models (Model 1: age, sex, estimated glomerular filtration rate [eGFR], and medicines for renal anemia; Model 2: Model 1 + antiplatelets; Model 3: Model 1 + comorbid conditions [hypertension, diabetes mellitus, hyperlipidemia, hyperuricemia, and history of ischemic heart disease]). A stratified analysis was conducted, dividing patients based on a hemoglobin concentration level of 11 g/dL. All statistical analyses were conducted using JMP 17 software (SAS Institute, Inc., Cary, NC, USA). Statistical significance was set at P < 0.05.

## Results

**Figure 1:**
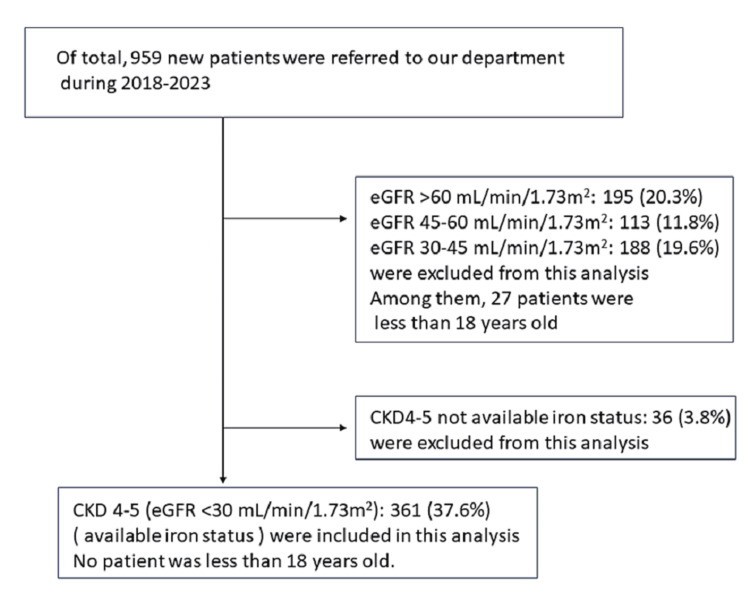
Patient flow

Among the 959 patients referred to our department between 2018 and 2023, 397 patients had stage 4 or 5 CKD. Iron-associated parameters were available for 361 patients (91%). Figure 1 illustrates the patient flow chart.

The mean age of the included patients was 76.8±12.1, and 54% of the patients were males. Nephrosclerosis, accounting for nearly half of all cases of iron deficiency, was the most prevalent cause of renal failure. Table 1 summarizes the patient's characteristics. According to the definition of iron deficiency, 169 patients (47%) had iron deficiency. Notably, these patients were older and female, and the prevalence of nephrosclerosis as the cause of renal failure was higher among them (Table 1).

**Table 1:** Demographic data of patients with or without iron deficiency Wilcoxon ranked sum test or chi-square test was used to analyze. P<0.05 was considered statistically significant. Data on hematuria were available (total: n=356, patients without iron deficiency: n=190, and patients with iron deficiency: n=166). Urinary protein/urinary creatinine was available (total: n=352, patients without iron deficiency: n=189, and patients with iron deficiency: n=163). NS: nephrosclerosis; ADPKD: autosomal dominant polycystic kidney disease; MCV: mean corpuscular volume; MCH: mean corpuscular hemoglobin, MCHC: mean corpuscular hemoglobin concentration; eGFR: estimated glomerular filtration rate; TSAT: transferrin saturation; UP/UCr: urinary protein/ urinary creatinine.

Variable	Total (N=361)	Patients without iron deficiency (n=192)	Patients with iron deficiency (n=169)	P-value
Age (years old)	76.8 ± 12.1	75.0 ± 12.2	78.9 ± 11.6	<0.001
Sex (male)	195, 54.0%	122, 63.5%	73, 43.2%	<0.001
Height (cm)	156.6 ± 10.4	158.8 ± 10.5	154.2 ± 9.7	<0.001
Weight (kg)	57.8 ± 13.9	59.9 ± 15.2	55.4 ± 11.8	0.003
Body mass index (kg/m^2^)	23.4 ± 4.3	23.6 ± 4.5	23.3 ± 4.1	0.54
Cause of renal failure				
NS (n, %)	148, 41.0%	71, 37.0%	77, 45.6%	0.18
Diabetic nephropathy (n, %)	114, 31.6%	63, 32.8%	51, 44.7%	
Glomerulonephritis (n, %)	46, 12.7%	30, 15.5%	16, 30.2%	
ADPKD (n, %)	4, 1.1%	2, 1.0%	2, 1.2%	
Others (n, %)	25, 6.9%	13, 6.8%	12, 7.1%	
Unknown (n, %)	24, 6.6%	13, 6.8%	11, 6.5%	
Comorbid conditions				
Diabetes mellitus (n, %)	143, 39.6%	78, 40.6%	65, 38.5%	0.65
Hypertension (n, %)	335, 92.8%	181, 94.3%	154, 91.1%	0.25
Hyperlipidemia (n, %)	143, 39.6%	72, 37.5%	71, 42.0%	0.38
Hyperuricemia (n, %)	146, 60.4%	88, 45.8%	58, 39.7%	0.03
Ischemic heart disease (n, %)	50, 13.9%	18, 9.4%	32, 18.9%	0.009
Smoking status				
Current (n, %)	55, 15.3%	39, 20.4%	16, 9.5%	<0.001
Past (n, %)	107, 29.7%	64, 33.5%	43, 25.4%	
Never (n, %)	198, 55.0%	88, 46.1%	110, 65.1%	
White blood cell (×10^3^/ μL)	6.4 ± 2.1	6.4 ± 1.9	6.5 ± 2.3	0.73
Red blood cell (×10^6^/ μL)	3.61 ± 0.71	3.59 ± 0.69	3.64 ± 0.72	0.75
Hemoglobin (g/dL)	10.8 ± 2.1	10.9 ± 2.1	10.7 ± 2.0	0.24
MCV (fL)	92.3 ± 6.6	93.0 ± 6.5	91.4 ± 6.6	0.049
MCH (pg)	30.2 ± 2.4	30.8 ± 2.2	29.6 ± 2.4	<0.001
MCHC (%)	32.6 ± 1.1	33.0 ± 1.1	32.3 ± 1.1	<0.001
Platelet (× 10^3^/μL)	205 ± 82	200 ± 81	211 ± 83	0.17
Blood urea nitrogen (mg/dL)	46.6 ± 21.0	49.0 ± 23.6	44.0 ± 17.2	0.12
Creatinine (mg/dL)	3.26 ± 1.97	3.56 ± 2.11	2.92 ± 1.75	<0.001
eGFR (ml/min/1.73 m^2^)	17.0 ± 7.0	16.2 ± 7.2	18.0 ± 6.7	0.02
Albumin (g/dL)	3.7 ± 0.6	3.7 ± 0.6	3.6 ± 0.6	0.50
Ferritin (ng/mL)	130 (58−239)	227 (152−336)	57 (31−86)	<0.001
TSAT (%)	27.5 ± 13.1	32.7 ± 12.1	21.6 ± 11.5	<0.001
Hematuria (yes) (n, %)	78, 21.9%	51, 26.8%	27, 16.3%	0.02
UP/UCr (g/gCr)	1.4 (0.3−4.4)	2.2 (0.6-5.1)	1.3 (0.2-3.3)	0.02

Sixty-one (17%) patients had an absolute iron deficiency. Table 2 summarizes the ferritin levels.

**Table 2:** Ferritin status in the patients with or without iron deficiency

Variables	Patients without iron deficiency (n=192)	Patients with iron deficiency (n=169)
Ferritin <50 ng/mL (n, %)	0, 0%	77, 45.6%
Ferritin 50-100 ng/mL (n, %)	0, 0%	64, 37.9%
Ferritin >100 ng/mL (n, %)	192, 100%	28, 16.6%

**Figure 2:**
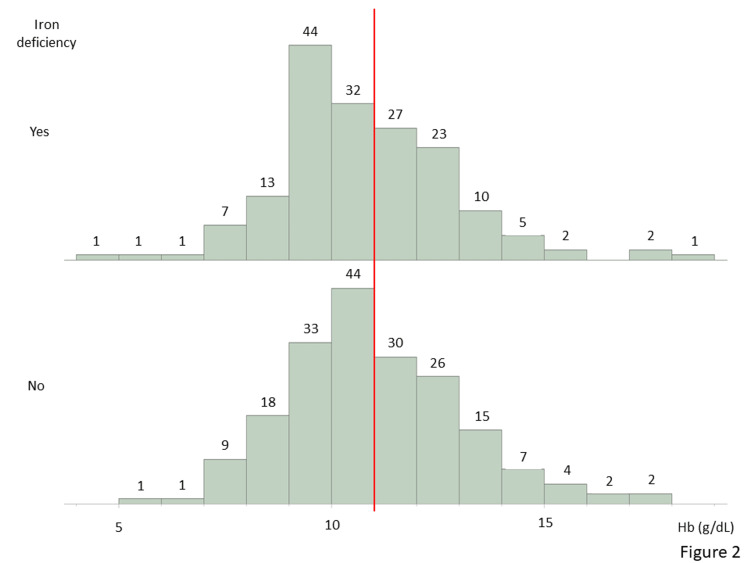
Histogram of hemoglobin in patients with and without iron deficiency The upper area shows patients with iron deficiency, and the lower area shows patients without iron deficiency.

Figure 2 presents hemoglobin histograms categorized according to the presence of iron deficiency. Ninety-nine (59%) of the 169 patients with iron deficiency had hemoglobin levels of <11 g/dL. In contrast, 106 (55%) of the patients without iron deficiency had hemoglobin levels of <11 g/dL. No significant differences were observed between patients with and without iron deficiency in terms of red blood cell counts and hemoglobin levels; however, the mean corpuscular volume, mean corpuscular hemoglobin, and mean corpuscular hemoglobin concentration were higher in the patients without iron deficiency (Table 1).

**Figure 3:**
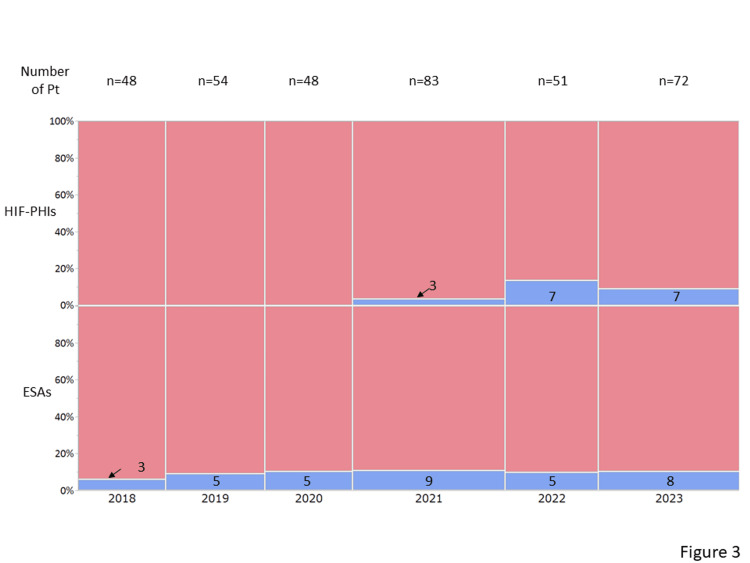
Annual change in the prescription rate for renal anemia Blue bars indicate the number of patients prescribed erythropoietin-stimulating agents or hypoxia-inducible factor prolyl-hydroxylase inhibitors.

**Figure 4:**
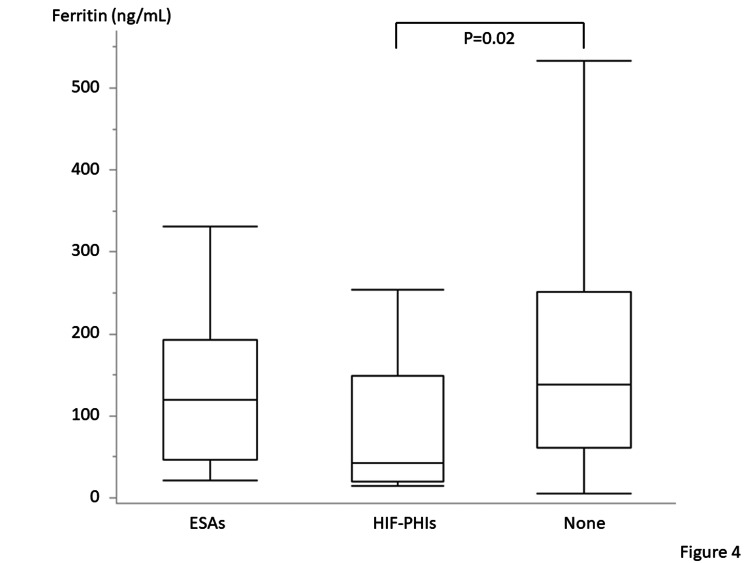
Serum ferritin levels of the patients receiving hypoxia-inducible factor prolyl-hydroxylase inhibitors (HIF-PHIs), erythropoietin stimulating agents (ESAs), and no medicines for renal anemia A significant difference is observed between the patients treated with HIF-PHIs and ESAs. Wilcoxon rank-sum test with a post-hoc Bonferroni correction was used. Statistical significance is set at P < 0.05.

**Figure 5:**
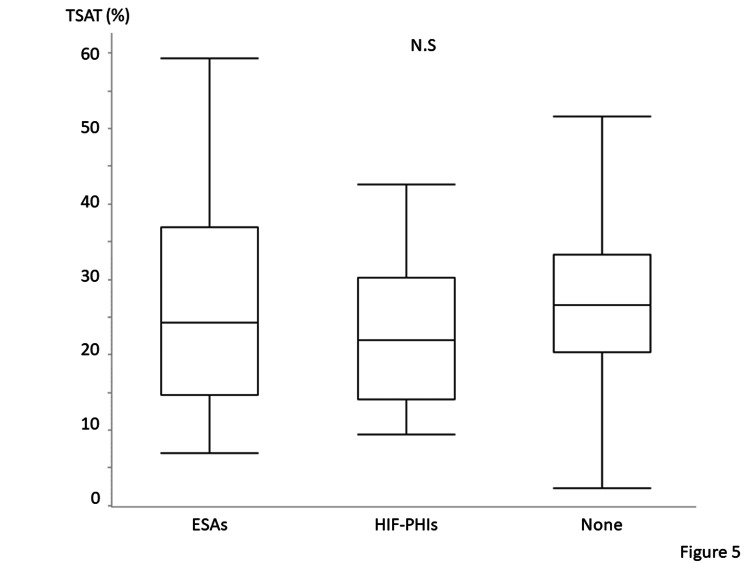
Transferrin saturation (TSAT) of the patients receiving hypoxia-inducible factor prolyl-hydroxylase inhibitors (HIF-PHIs), erythropoietin stimulating agents (ESAs), and no medicines for renal anemia No significant differences are observed among the three groups. Wilcoxon rank-sum test with a post-hoc Bonferroni correction was used. Statistical significance is set at P < 0.05.

Table 3 presents the medications used for the treatment of CKD. No significant difference except in the antiplatelets was observed between patients with iron deficiency and those without iron deficiency. Approximately 10% of the included patients receive oral iron supplementation. Ferrous citrate was prescribed in 31 patients, and ferrous sulfate was prescribed in three patients. No patient was administered intravenous iron regularly. Among the 35 patients prescribed ESAs, 25 and 10 patients were prescribed darbepoetin and epoetin beta pegol, respectively, at their primary facilities. Among the 17 patients who were prescribed HIF-PHIs, daprodustat, vadadustat, roxadustat, and molidustat were prescribed to six, five, three, and three patients, respectively. No significant differences were observed between patients with and without iron deficiency in terms of the number of patients administered medications for renal anemia, such as ESAs and HIF-PHIs. Annual changes in medications for renal anemia are shown in Figure 3. Figure 4 shows the lowest ferritin levels (median 42 [IQR: 20-147]) in patients who received HIF-PHIs compared with those who received ESAs (median 120 [IQR: 46-193], P=0.20) and those who did not receive any medications for renal anemia (median 139 [IQR: 61-251], P=0.02). However, no significant differences were observed among these three patient groups in terms of TSAT (Figure 5). Thirty-four patients received oral iron supplements; however, no significant differences were observed between patients who did and did not receive iron supplements in terms of ferritin levels, TSAT, or the prevalence of iron deficiency (data not shown). Patients with ischemic heart disease tend to be prescribed antiplatelets. Among 50 patients with a history of ischemic heart disease, 31 patients (62%) were prescribed antiplatelets. On the other hand, the prescription rate of antiplatelets in patients without a history of ischemic heart disease was lower (21%) (P<0.001).

**Table 3:** Summary of medicines for treating chronic kidney diseases at the first visit of the nephrology department Wilcoxon ranked sum test or chi-square test was used to analyze. P<0.05 was considered statistically significant. ARBs: angiotensin II receptor blockers; ACE: angiotensin-converting enzyme; MRA: mineral corticoid antagonists; ESA: erythropoietin stimulating agents; HIF-PHI: hypoxia-inducible factor prolyl hydroxylase inhibitors; SGLT2: sodium-glucose cotransporter-2; PPIs: proton-pump inhibitors.

Variable	Total (N=361)	Patients without iron deficiency (n=192)	Patients with iron deficiency (n=169)	P-value
ARBs (n, %)	160, 44.3%	85, 44.3%	75, 44.4%	0.98
ACE inhibitors (n, %)	18, 5.0%	9, 4.7%	9, 5.3%	0.78
MRAs (n, %)	40, 11.0%	18, 9.4%	22, 13.0%	0.27
Oral iron supplement (n, %)	34, 9.4%	14, 7.3%	20, 11.8%	0.14
ESA (n, %)	35, 9.7%	16, 8.3%	19, 11.2%	0.35
HIF-PHIs (n, %)	17, 4.7%	6, 3.1%	11, 6.5%	0.13
Medicines for renal anemia (ESA or HIF-PHIs) (n, %)	52, 14.4%	22, 11.5%	30, 17.8%	0.09
SGLT2 inhibitors (n, %)	27, 7.4%	16, 8.3%	11, 6.5%	0.51
PPIs and H2 blockers	119, 33.0%	57, 29.7%	62, 36.7%	0.16
Antiplatelets	97, 26.9%	43, 22.4%	54, 32.0%	0.04
Anticoagulants	46, 12.7%	22, 11.5%	24, 14.2%	0.44

A multivariable logistic regression analysis for iron deficiency was performed using two models. Age, sex, body mass index (BMI), history of diabetes mellitus, eGFR, and medications for renal anemia (ESA and HIF-PHIs) were included as covariates in Model 1. Antiplatelets were included in Model 2 in addition to all the variables from Model 1. All variables from Model 1 and comorbid conditions were included as covariates in Model 3. Model 1 indicated that female sex, eGFR, and medications for renal anemia were associated with iron deficiency. Models 2 and 3 exhibited similar tendencies; however, antiplatelet and ischemic heart disease were associated with iron deficiency, respectively. Table 4 presents the results of the multivariable analysis.

**Table 4:** Multivariable logistic regression analysis for iron deficiency OR: odds ratio, 95% CI, 95% confidence interval; BMI: body mass index; eGFR: estimated glomerular filtration rate; IHD: ischemic heart disease. Multivariable logistic analysis was used. P<0.05 was considered statistically significant.

Variables	Model 1			Model 2			Model 3		
	OR	95% CI	P-value	OR	95% CI	P-value	OR	95% CI	P-value
Age	1.02	1.00−1.04	0.02	1.02	1.00−1.04	0.047	1.02	1.00−1.04	0.03
Sex (female)	2.18	1.39−3.42	<0.001	2.36	1.49−3.73	<0.001	2.05	1.28−3.30	0.003
BMI	1.01	0.96−1.06	0.71	1.01	0.96−1.07	0.70	1.02	0.96−1.07	0.55
eGFR	1.05	1.02−1.09	0.002	1.05	1.02−1.09	0.002	1.04	1.01−1.08	0.01
Medicines for renal anemia	1.98	1.05−3.75	0.03	2.08	1.09−3.95	0.03	1.98	1.04−3.79	0.04
Antiplatelets	-	-	-	1.76	1.07−2.91	0.03	-	-	-
Hypertension	-	-	-	-	-	-	0.69	0.27−1.75	0.44
Diabetes mellitus	-	-	-	-	-	-	1.02	0.63−1.65	0.94
Hyperlipidemia	-	-	-	-	-	-	1.02	0.63−1.64	0.94
Hyperuricemia	-	-	-	-	-	-	0.73	0.45−1.17	0.19
IHD	-	-	-	-	-	-	2.22	1.12−4.40	0.02

Stratified analysis for the same logistic regression models was conducted by dividing patients based on a hemoglobin concentration level of Hb 11 g/dL. In patients with hemoglobin concentrations <11 g/dL, antiplatelets had a negative impact on iron deficiency. On the other hand, in patients with hemoglobin concentrations ≥11 g/dL, medicines for renal anemia and ischemic heart disease have negative impacts on iron deficiency. The results are shown in Tables 5-6.

**Table 5:** Multivariable logistic regression analysis for iron deficiency in patients with hemoglobin levels less than 11 g/dL OR: odds ratio, 95% CI, 95% confidence interval; BMI: body mass index; eGFR: estimated glomerular filtration rate; IHD: ischemic heart disease. Multivariable logistic analysis was used. P<0.05 was considered statistically significant.

Variables	Model 1			Model 2			Model 3		
	OR	95% CI	P-value	OR	95% CI	P-value	OR	95% CI	P-value
Age	1.02	0.99−1.04	0.20	1.02	0.99−1.04	0.25	1.02	0.99−1.05	0.16
Sex (female)	1.97	1.09−3.57	0.02	2.17	1.18−4.00	0.01	1.74	0.93−3.24	0.08
BMI	1.05	0.97−1.14	0.25	1.06	0.97−1.15	0.18	1.05	0.97−1.14	0.22
eGFR	1.07	1.02−1.11	0.005	1.06	1.02−1.11	0.007	1.06	1.01−1.11	0.02
Medicines for renal anemia	1.36	0.62−2.96	0.44	1.40	0.64−3.07	0.40	1.32	0.60−2.90	0.49
Antiplatelets	-	-	-	1.76	1.07−2.91	0.01	-	-	-
Hypertension	-	-	-	-	-	-	0.81	0.27−2.44	0.70
Diabetes mellitus	-	-	-	-	-	-	0.99	0.52−1.89	0.99
Hyperlipidemia	-	-	-	-	-	-	1.08	0.56−2.06	0.82
Hyperuricemia	-	-	-	-	-	-	0.61	0.32−1.16	0.13
IHD	-	-	-	-	-	-	1.57	0.61−4.01	0.34

**Table 6:** Multivariable logistic regression analysis for iron deficiency in patients with hemoglobin levels 11 g/dL and over OR: odds ratio, 95% CI, 95% confidence interval; BMI: body mass index; eGFR: estimated glomerular filtration rate; IHD: ischemic heart disease. Multivariable logistic analysis was used. P<0.05 was considered statistically significant.

Variables	Model 1			Model 2			Model 3		
	OR	95% CI	P-value	OR	95% CI	P-value	OR	95% CI	P-value
Age	1.03	0.997−1.06	0.07	1.03	0.99−1.06	0.11	1.02	0.99−1.06	0.16
Sex (female)	2.30	1.09−4.85	0.03	2.40	1.12−5.15	0.02	2.57	1.16−5.71	0.02
BMI	0.99	0.92−1.07	0.84	0.99	0.92−1.06	0.70	1.00	0.92−1.08	0.97
eGFR	1.04	0.99−1.09	0.15	1.04	0.99−1.09	0.14	1.04	0.98−1.09	0.18
Medicines for renal anemia	4.39	1.23−15.25	0.01	4.54	1.30−15.87	0.02	5.13	1.37−19.17	0.009
Antiplatelets	-	-	-	1.22	0.56−2.68	0.61	-	-	-
Hypertension	-	-	-	-	-	-	0.93	0.13−6.66	0.94
Diabetes mellitus	-	-	-	-	-	-	1.09	0.51−2.33	0.82
Hyperlipidemia	-	-	-	-	-	-	0.85	0.40−1.81	0.67
Hyperuricemia	-	-	-	-	-	-	0.87	0.41−1.86	0.73
IHD	-	-	-	-	-	-	3.38	1.18−9.71	0.02

Nephrologists at our facility conducted iron supplementation for 27 patients (16.0%) with iron deficiency and 5 patients (2.6%) without iron deficiency within three months from the first visits (ferrous citrate [n=28], ferric citrate [n=1], and intravenous iron supplementation [n=3]). A significant difference was observed between the groups (P<0.001). On the other hand, ESAs or HIF-PHIs were prescribed in 49 patients (25.5%) without iron deficiency and 30 patients (17.8%) with iron deficiency (Darbepoetin [n=41], continuous erythropoiesis receptor activation [n=37], and daprodustat [n=1]). No significant difference was observed (P=0.10).

## Discussion

This was a cross-sectional retrospective analysis of newly referred patients with stage 4 or 5 CKD at a single center. Among the 361 patients screened, 205 (57%) had hemoglobin levels of <11 g/dL. Renal anemia was considered the most likely cause of anemia; however, iron deficiency was detected in 99 patients with hemoglobin levels of <11 g/dL at the time of the initial referral. Iron supplementation should be considered the first step to treating anemia in such cases. Less than 15% of the patients were prescribed medications for renal anemia by the primary physicians. Furthermore, no significant differences were observed between patients with and without medications for renal anemia in terms of the iron indices, except for the ferritin levels between patients receiving HIF-PHIs and patients who were not receiving any medications for renal anemia. Multivariable logistic regression analysis revealed that age, female sex, eGFR, medications for renal anemia, and a history of ischemic heart disease were associated with iron deficiency.

According to the Japanese Chronic Kidney Database, approximately 40% of individuals with stage 4 CKD had hemoglobin levels of <11 g/dL; in contrast, nearly half of the non-dialysis-dependent patients with stage 5 CKD had hemoglobin levels of <11 g/dL [8]. ESAs or HIF-PHIs were not administered to the majority of patients included in this study, and the prevalence of the patients whose hemoglobin levels should be higher than in previous reports. No evidence of anemia was observed in 70 patients (41%) with iron deficiency; however, a previous study showed that the prevalence of iron deficiency without anemia was high in patients with CKD [9].

Iron supplementation is recommended for patients with CKD exhibiting iron deficiency per the CKD guidelines [6,10-12]. Multivariable logistic regression analysis revealed that iron deficiency is associated with the use of medications for renal anemia, indicating that patients receiving medication for renal anemia are more susceptible to developing iron deficiency. Although <15% of patients were prescribed medications for renal anemia at previous clinics, routine assessment of iron status in patients with renal failure and prescription of ESAs or HIF-PHIs for those with renal anemia were performed at our institution. Iron supplementation was also considered if an iron deficiency was detected. HIF-PHIs are particularly convenient for use in primary clinics as they can be administered orally, which facilitates easy dose adjustments and prescriptions. Moreover, they enhance iron consumption and effectively address renal anemia [1]. However, the iron levels must be monitored carefully following treatment with HIF-PHI, as the ferritin levels in patients receiving HIF-PHI are lower than those in patients not receiving any medications for renal anemia. Nevertheless, some concerns remain despite the convenience of HIF-PHIs. Several meta-analyses have reported the absence of any significant differences between patients treated with HIF-PHIs and ESAs in terms of the incidence of adverse events [13,14]; however, a pooled analysis revealed that vadadustat could increase the risk of major adverse cardiovascular events compared with that of darbepoetin [15]. Thus, careful monitoring of the iron status in patients receiving HIF-PHIs is imperative, as HIF-PHIs accelerate iron use in the body.

The prevalence of cardiovascular disease (CVD) is higher in patients with CKD than in the general population [9]. Given its critical nature, the prevention of CVD is a substantial factor in the treatment of CKD. Iron deficiency is considered to be associated with CVD, regardless of anemia status [9]. Iron deficiency increases the risk of thromboembolism, underscoring the importance of managing and preventing iron deficiency. A higher number of patients with ischemic heart disease were present in the iron-deficiency group in this study. A previous report on patients with CKD revealed that patients with coronary artery disease tended to have iron deficiency [16,17]. Another study revealed that patients with coronary artery disease tended to have iron deficiency [7]. Patients undergoing percutaneous coronary intervention often receive antiplatelet therapy, which increases the risk of gastrointestinal bleeding [16,17]. In fact, patients with ischemic heart disease tended to be prescribed antiplatelets in this study, which might have played an important role in iron deficiency. However, due to strong collinearity, ischemic heart disease and antiplatelets could not be included simultaneously in the multivariate logistic regression model. Poor cardiac output hinders iron absorption in the gastrointestinal tract [16,17]. Thus, iron deficiency worsens cardiac function when patients with a history of ischemic heart disease develop heart failure, resulting in a vicious cycle [17]. Patients with co-existing CKD and iron deficiency without anemia generally do not receive treatment; however, iron supplementation improves the life prognosis if heart failure is detected [18]. Nevertheless, these patients have a higher risk of developing recurrent ischemic heart disease, emphasizing the need to prevent iron deficiency, particularly in patients with a history of ischemic heart disease.

The present study has certain limitations. First, the background characteristics of the newly referred patients may have introduced bias as this was a single-center study. Moreover, the follow-up data on iron status were not accessible as this was a cross-sectional study. We cannot evaluate whether patients had heart failure at the first referral because we did not perform chest X-ray imaging on all the patients. We could merely estimate heart failure based on the history of ischemic heart disease in clinical history. The iron status was assessed during the initial visit, and regular monthly monitoring was uncommon unless patients received oral iron supplementation. Additionally, patients on iron supplementation could be iron deficient; however, reverse causality is possible because this analysis was conducted as a cross-sectional study. Therefore, we merely differentiated among the included patients whether iron deficiency was present or not based on their iron status at the first referral. Nephrologists at our facility tended to perform iron supplementation in anemic patients with iron deficiency, and they prescribed ESAs and HIF-PHIs in patients with anemia. However, they sometimes suggested iron supplementation or the prescription of ESAs and HIF-PHIs to primary physicians. Therefore, the actual prescription rate for these medicines is unknown. We cannot assess other factors associated with anemia, such as vitamin B12 and folate levels, because these data were lacking in nearly all the cases. Lastly, the increase in the number of new patients with stages 4 or 5 CKD in 2021 observed in the present study may be attributed to the ongoing coronavirus pandemic.

## Conclusions

In the present study, nearly half of the patients presented with iron deficiency at the time of the initial presentation at the nephrology department. Medications for renal anemia had been prescribed to some of these patients at other clinics, and these patients tended to have iron deficiency. Although various agents are available for the treatment of renal anemia, iron status must be assessed before prescribing them to patients with renal failure. Iron deficiency can be linked to refractory anemia and cardiovascular diseases; thus, patients with CKD must be treated with caution.
